# Focused Study on the Quiet Side Effect in Dwellings Highly Exposed to Road Traffic Noise

**DOI:** 10.3390/ijerph9124292

**Published:** 2012-11-22

**Authors:** Timothy Van Renterghem, Dick Botteldooren

**Affiliations:** Acoustics Research Group, Department of Information Technology (INTEC), Ghent University, Sint-Pietersnieuwstraat 41, Gent B-9000, Belgium; Email: dick.botteldooren@intec.ugent.be

**Keywords:** road traffic noise, noise annoyance, quiet side, noise-induced sleep disturbance, noise sensitivity

## Abstract

This study provides additional evidence for the positive effect of the presence of a quiet façade at a dwelling and aims at unraveling potential mechanisms. Locations with dominant road traffic noise and high L_den_-levels at the most exposed façade were selected. Dwellings both with and without a quiet façade were deliberately sought out. Face-to-face questionnaires (N = 100) were taken to study the influence of the presence of a quiet side in relation to noise annoyance and sleep disturbance. As a direct effect, the absence of a quiet façade in the dwelling (approached as a front-back façade noise level difference smaller than 10 dBA) leads to an important increase of at least moderately annoyed people (odds-ratio adjusted for noise sensitivity equals 3.3). In an indirect way, a bedroom located at the quiet side leads to an even stronger reduction of the self-reported noise annoyance (odds-ratio equal to 10.6 when adjusted for noise sensitivity and front façade L_den_). The quiet side effect seems to be especially applicable for noise sensitive persons. A bedroom located at the quiet side also reduces noise-induced sleep disturbances. On a loud side, bedroom windows are more often closed, however, conflicting with the preference of dwellers.

## 1. Introduction

The World Health Organization (WHO) “*Burden of disease by environmental noise*” report [1] quantifies the negative effects of long-term exposure to road traffic noise levels. Estimates in terms of “disability-adjusted life-years” (DALYs) list sleep disturbance, annoyance, ischemic heart diseases, cognitive impairment of children and tinnitus as major health effects, leading to a loss of about one million healthy life years every year in western Europe. The first round of strategic noise mapping in the European Union showed that within the big urban agglomerations (with more than 250,000 inhabitants) about 56 million people are exposed to L_den_ levels above 55 dBA, and 40 million to L_night_ levels above 50 dBA, resulting from road traffic [2]. Outside agglomerations, near major roads, these numbers are 34 million and 25 million for L_den_ and L_night_, respectively [2]. In addition, noise pollution is a major source of complaints [1], especially in densely populated areas. Repeated extensive measurement campaigns in the period 1996–2009 in Flanders (Belgium) hardly showed any improvement in the noise exposure measured as equivalent levels in front of the façade [3]. As a result, it can be concluded that road traffic noise is a persistent and major environmental problem.

In many urban situations, front façade noise levels are hard to reduce, unless drastic and mainly traffic-related measures are taken (strongly reducing traffic intensity, changing traffic composition, e.g., by banning heavy vehicles, or reducing vehicle speed). Especially in urban streets and so-called street canyons, there is a strong amplification of the noise levels by multiple reflections in between façades, consisting of mostly rigid materials. This amplification affects both front and back façade noise levels [4]. Measures limiting sound intensity during propagation are often difficult to apply, especially in a dense urban setting.

Therefore, another possible strategy to reduce the impact of exposure to excessive road traffic noise is increasingly gaining attention in recent years: providing a quiet façade or a quiet courtyard at a dwelling [5,6,7]. The presence of a quiet façade could create a possible way of coping for the inhabitant of a dwelling exposed to high noise levels at the most exposed façade: a quiet side allows residents to escape from excessive noise levels depending on their activities, or to assign rooms in a dwelling for particular use (e.g., a bedroom at the quiet side of the house). Exploiting the quiet side effect could be an attractive, and practical measure to take into account in city planning. Clearly, there are some limits as for the maximum levels at the most-exposed façade where the positive effect of a quiet side on reported noise annoyance can still be expected, as discussed e.g., in [5].

The positive effect of the presence of a quiet side as for noise perception has been shown before by means of a number of surveys. Ohrstrom *et al*. [5] found by means of a written questionnaire (N = 956) that access to a quiet side of one’s dwelling reduces disturbances (annoyance, disturbed daytime relaxation, sleep, and decreased physiological and psychological well-being) by an average of 30–50%. This corresponds to a reduction in sound level at the most-exposed side near 5 dBA (using L_Aeq,24h_). Ohrstrom *et al*. [5] concluded that in order to protect 80% of the people from both annoyance and other adverse noise-related effects, sound levels from road traffic should not exceed 60 dBA at the most-exposed side, in combination with a quiet side below 45 dBA.

De Kluizenaar *et al*. [7] showed by means of a postal questionnaire (N = 18,000) in the city of Eindhoven (The Netherlands) that noise annoyance was less likely for the subgroup with a relatively quiet façade (level difference between most and least exposed façade, Q, larger than 10 dBA) as compared to dwellings with a smaller level difference (Q < 10 dBA). The difference in response between these two groups increased with higher most exposed façade noise levels (using L_den_-values) and with increasing Q. In the category with a median L_den_ level near 65 dBA (most exposed façade), the odds ratio for the noise annoyance response (dichotomous, annoyed versus not annoyed, see [7]) reduced from 8.0 to 6.5 with an increase in Q of about 10 dBA.

Gidlof-Gunnarsson *et al*. [8] showed that the environmental physical quality of a quiet side, specifically for courtyards, is an important modifier. Access to an attractive quiet courtyard (defined as “natural” and “useable”) is associated with less noise annoyance and noise-disturbed outdoor activities for residents. The percentage of respondents reporting at least moderate annoyance in the sound level interval 63–68 dBA (L_Aeq,24h_ at the most exposed façade) were 42% and 29%, for low quality and high quality courtyards, respectively.

Shepherd *et al*. [9] discussed that noise annoyance and sleep disturbance are the most important mechanisms leading to noise-induced health deficits. Therefore, both effects are considered here and their association with the quiet side effect is studied.

In addition, noise sensitivity is also included. Noise sensitivity was shown before to be a strong predictor of noise annoyance and reported sleep disturbance. Stansfeld [10] concluded that noise sensitivity is a stable personal trait. Miedema *et al*. [11] found in their meta-analysis that the influence of noise sensitivity on noise annoyance becomes especially important at higher exposure levels. Similarly, Lercher [12] found that reported sleep disturbance by road traffic noise was not related to noise sensitivity at low noise levels, while at higher noise levels the percentage of noise sensitive persons reporting sleep disturbance was much higher. Miedema *et al*. [13] found that the difference in annoyance between non-sensitive and highly sensitive persons (categorization in three groups) could be equivalent to a level difference of 11 dBA. For health-related effects like hypertension and ischemic heart disease, noise sensitivity was even considered to be the only relevant parameter by Fyhri *et al*. [14]; in their analyses, no relationship with noise exposure could be identified.

Noise-induced sleep disturbance is considered to be the most serious health effect of exposure to environmental noise [15]. The location of the bedroom in a dwelling is therefore an important parameter in relation to the quiet side concept. Lercher [16] indicated that sleeping room orientation relative to the noise source is expected to be an important moderating effect. Ohstrom [5] concluded that only when the bedroom window faces the quiet side of a dwelling, WHO guidelines for undisturbed sleep [15] could be reached. The benefit of access to a quiet side specifically for sleep was estimated between 8 and 18% based on their survey [5]. Paunovic *et al*. [17] stated that relevant predictors of high annoyance in a noisy street were the orientation of the bedroom, noise annoyance experienced at the workplace, and noise sensitivity. Near quiet streets, the orientation of the bedroom did not show to be a relevant parameter, while noise sensitivity was the most important one. Not only the location of the bedroom, but also habits related to opening or closing windows could be relevant, and specific questions on this were included in the current study.

This study aims at providing additional evidence on the effect of a quiet dwelling façade in an urban and suburban environment and tries to unravel potential mechanisms explaining this effect. This study is concerned with road traffic noise, as this is the most widespread environmental noise source in the region under study [3]. We opted for a face-to-face questionnaire (interview) since it allows gathering more detailed information as discussed further in this article. The study focuses on two major effects, namely noise annoyance and sleep disturbance. Given the potential importance of noise sensitivity, the latter was assessed as well by additional questions. A face-to-face questionnaire comes however at the cost of having less respondents since more effort is needed in gathering data. Therefore, the respondents have been selected in specific zones based on noise exposure and presence or absence of a quiet side.

## 2. Methodology

### 2.1. Participant Selection

Participants from nine neighborhoods in the city of Ghent (Belgium) were selected, including inner city zones and suburban areas. The choice of these neighborhoods was based on specific requirements regarding the most and least exposed façade of dwellings that will be explained in the next section. Participants were directly contacted, without prior announcement, by knocking on doors at the selected sites. Interested people who had no time at the moment of the visit were offered a second visit. The minimum age for respondents was 18 years.

A face-to-face questionnaire was performed and 100 participants were aimed at. There was a single interviewer. The survey was announced as general research concerning the living environment, and how it was affected by the architecture of their dwelling. It was stressed that people satisfied with their living environment should participate too. An important prerequisite to start the questionnaire was that participants had lived at least 1 year at the current location, in order to prevent people making a comparison relative to their previous dwelling (to prevent so-called “change effects” in addition to the “exposure effect”, see e.g., Reference [18]), and to ensure that participants were sufficiently familiar with the local noise climate.

### 2.2. Noise Exposure Assessment

**Figure 1:**
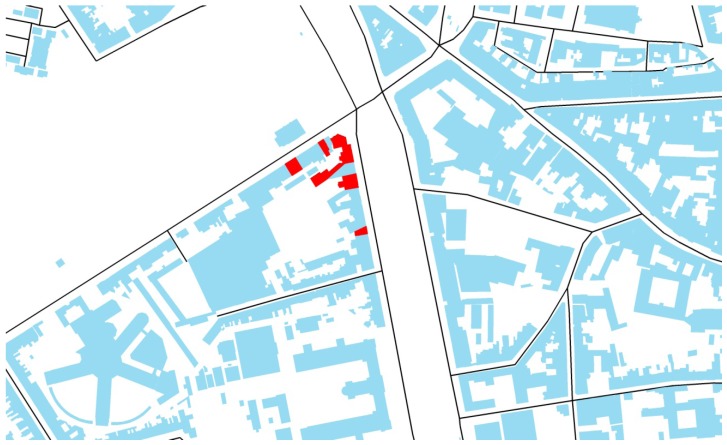
GIS (geographic information system) building layer showing a neighborhood where a clear quiet side was present. The dwellings where interviews were taken are the red-colored buildings.

**Figure 2:**
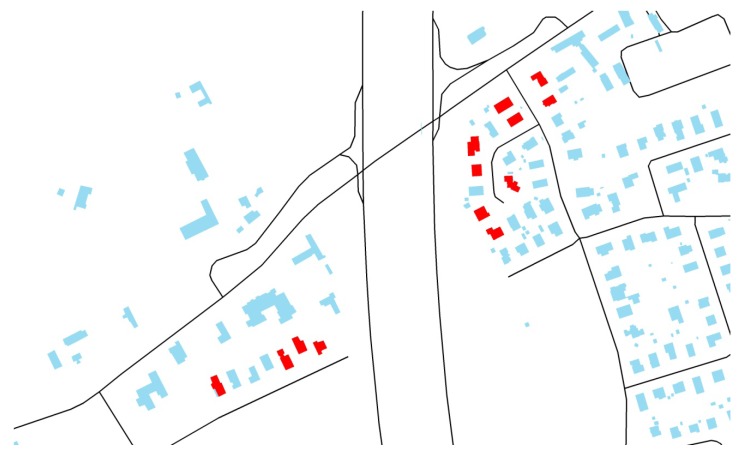
See Figure 1, but now for a neighborhood where a quiet side was absent.

The noise exposure at the most exposed façade was extracted from the road traffic noise map approved by the Flemish regional government for the agglomeration of Ghent, which has been reported to the European Commission in the framework of the Environmental Noise Directive (END) [19]. Locations dominated by road traffic were selected, where the most exposed façade fell in the classes 65–75 dBA for day-evening-night corrected (yearly) equivalent sound pressure levels (= L_den_). About half of the neighborhoods were selected with dwellings having a clearly shielded façade (as illustrated in Figure 1), the other half with no quiet side at all (see Figure 2). The presence of a quiet side was assessed based on the noise map, in combination with analyzing building geometry and their surroundings using aerial photographs, and by site visits. This selection procedure assured that the quiet side was accessible (windows, balcony, *etc*.) by the inhabitants of the dwelling and that there were no disturbances that are generally not included in END noise maps such as parking lots and ventilation units.

Road traffic noise levels at the most and least exposed façade were manually selected from the noise maps at the survey locations and include a quality check of the exposure data. Since noise levels at the most exposed façade in streets are strongly source driven, their correctness is generally considered to be reasonable; only very strong deviations from the actual traffic intensity or composition would lead to significant errors in the predicted levels. For less trafficked roads, such predictions in noise maps are less reliable. However, in the current study, only façades along (very) busy roads have been selected to reach the 65–75 dBA L_den_ exposure range. At shielded façades, more complex sound propagation aspects need to be taken into account when calculating noise maps. These are unfortunately often neglected to limit the computational cost. Typically, this leads to an underprediction of levels at the least-exposed façade. Therefore, only a rough classification regarding level difference between the loudest and quietest side will be used during analysis.

In current urban noise mapping research, engineering models are under development to account for these inaccurate predictions at shielded locations. An interesting concept called “background noise mapping” is discussed in Reference [20].

### 2.3. Survey Description

In a first part, some general questions were posed regarding the quality of living in the neighborhood, and annoyances caused by common environmental stressors. The first question looked at the general satisfaction regarding the quality of living in the neighborhood of the dweller, with indication of some examples of parameters to be taken into account (e.g., safety, child-friendly, environment, ...). A 5-point categorical scale was offered, with the following textual descriptions: “very satisfied”, “satisfied”, “more or less satisfied”, “not satisfied”, and “not at all satisfied”. Next, it was asked if the respondent would advise friends or relatives to come live in their neighborhood when considering the quality of the living environment. A 3-point categorical scale was offered (“yes”, “no”, “undecided”). Then, the ISO-standardized question [21] was asked regarding annoyance by noise, odor and light (“If you consider the past 12 months, to what degree were you annoyed or not annoyed by the following sources”). A 5-point categorical scale (“not at all annoyed”, “slightly annoyed”, “moderately annoyed”, “strongly annoyed”, and “extremely annoyed”) was proposed for each of these types of environmental nuisances.

In a second part, more detailed information about possible sources of noise annoyance was sought. These sources are traffic (“road”, “railway”, “air”, “water”), industry and small and medium enterprises (“delivery of goods by trucks”, “construction noise”, “industrial plants”, “trade and services”), leisure activities (“music from pubs, bars, and restaurants”, “music in cars”, “outdoor concerts”, “sports activities”, “street racing”), agricultural activities, neighbors (“children playing”, “animals”, “do-it-yourself home improvement”, “loud music/television”, “gardening”, “heating, ventilation and air conditioning units”), others. For each defined subcategory of the different sources of potential noise annoyance, the same scale was used as for the general noise annoyance question as discussed in previous paragraph.

In a third part, specific questions were asked regarding possible sleep disturbance by noise. Firstly, the interviewer made, with help of the respondent, a plan of the dwelling indicating the location of the different rooms. The interviewer assessed whether the bedroom was located at a quiet side of the dwelling (without further interaction with the dweller). In this way, no hints on the link between bedroom location and noise exposure were given. The number of hours of sleep per night was asked for. It was asked whether the respondent is hindered by road traffic noise during falling asleep, and if road traffic noise leads to awakening at night. A 4-point categorical answering scale (“never”, “sometimes”, “often”, “always”) was used. Specific questions were asked regarding opening or closing of the window of the bedroom: “Do you leave your bedroom window open (“always”, “never”, “only during summertime”)?”, “Do you like sleeping with open windows (“yes”, “no”)?”, “Do you close your bedroom window because of road traffic noise (“never”, “sometimes”, “often”, “always”)?”. Closing windows was shown to be an important way of coping before, influencing annoyance ratings [22].

In a fourth part, noise sensitivity was assessed using a Dutch adaptation of Weinstein’s noise-sensitivity scale [23], used previously in large-scale Flemish quality-of-life studies. This part contained 10 questions, and used a 6-point categorical scale with textual indication of the endpoints (“totally agree” and “totally disagree”). Finally, some general questions were asked about gender, age, education, professional activities, and the number of years living at the current location.

### 2.4. Statistical Analysis

Given the limited amount of respondents in the dataset, classification has been performed in order to have sufficient occurrences in the different cells when using frequency tables. The Chi-square test has been applied to check dependence between variables. The null hypothesis states that variables are independent. In order to have a clear dependence, this null hypothesis must be rejected with a sufficient degree of certitude (= 1 − *p*). Odds Ratios (OR) have been considered, and logistic regression is used to predict confidence intervals. Logistic regression with a dichotomous outcome (true or false) has been used, based on both continuous and dichotomous independent variables. In order to be statistically sound, 95% confidence intervals of OR should not contain 1. Effect modifiers have been looked for by multiple logistic regression. Statistical significance of model deviance reduction when including additional variables has been checked by likelihood ratio testing (based on the Chi-square distribution). The Matlab^®^ statistics toolbox has been used to perform all analyses.

## 3. Results and Discussion

### 3.1. Respondents Characteristics

**Figure 3:**
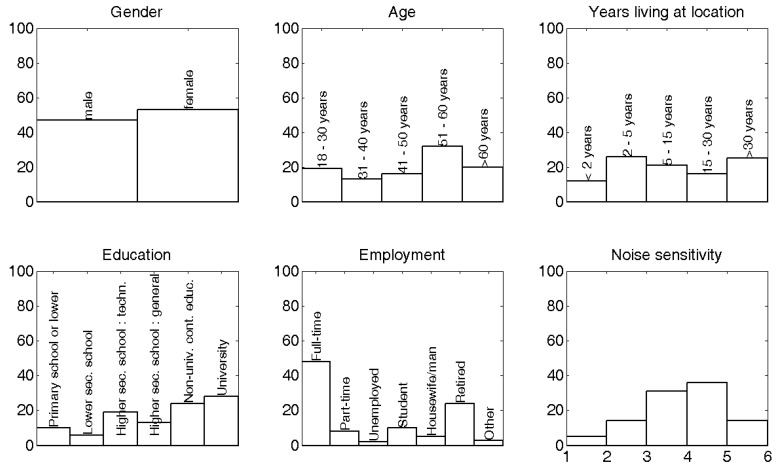
Distribution of answers of respondents related to gender, age, years of living at the current location, education, employment status and noise sensitivity. Noise sensitivity uses a continuous scale between 1 (= “not at all sensitive to noise”) and 6 (= “highly sensitive to noise”) and is based on a set of 10 questions.

The total number of respondents was 100. The willingness to participate was high and equaled 70%. From these, 4% was discarded since these inhabitants had been living less than one year at their current location. The distribution over gender, age, years living at the location, education, employment and noise sensitivity is shown in Figure 3. 52% of the respondents are more than 50 years old. 53% of the respondents were female. 52% of the respondents received a higher education (combining “non-university continued education” and “university”). The median of the noise sensitivity, after linearly averaging the responses on the 10 sensitivity questions, was at 3.9 (with 1 meaning “not at all sensitive to noise”, and 6 meaning “highly sensitive to noise”). No rectification to match the Flemish population has been performed on the data. An overview of the respondent characteristics, organized in front-back level difference intervals, is shown in Table 1.

**Table 1:** Overview of the dwelling and respondent characteristics, together with the answers to the annoyance and sleep related questions, distributed over 3 front-back level difference (Q) classes. The number of respondents is given in each category (N=100).

		Q < 10 dBA	10 dBA ≤ Q <20 dBA	Q ≥ 20 dBA
front façade level L_f_	L_f_ < 65 dBA	14	2	2
	65 dBA ≤ L_f_ < 75 dBA	22	29	18
	L_f_ ≥ 75 dBA	2	3	8
back façade level L_b_	L_b_ < 55 dBA	1	14	28
	55 dBA ≤ L_b_ < 65 dBA	21	17	0
	L_b_ ≥ 65 dBA	16	3	0
gender	male	22	16	9
	female	16	18	19
age	below 50	15	18	15
	above 50	23	16	13
years living at location	less than 5 years	6	23	9
	between 5 and 15 years	8	8	5
	more than 15 years	24	3	14
higher education	no	24	11	13
	yes	14	23	15
employment	full-time	17	19	12
	student	3	3	4
	retired	13	5	6
	part-time, unemployed and housewife/man	5	7	6
noise sensitivity	not sensitive (<3.5)	13	7	10
	sensitive (≥3.5)	25	27	18
neighborhood quality	“not” and “not at all” satisfied	7	1	2
	at least more or less satisfied	31	33	26
recommend neighborhood	“yes”	24	31	18
	“no” or “undecided”	14	3	10
noise annoyance	“not at all” and “slightly” annoyed	18	23	21
	at least moderately annoyed	20	11	7
odour annoyance	“not at all” and “slightly” annoyed	30	33	26
	at least moderately annoyed	8	1	2
bedroom at a quiet side	yes	3	7	10
	no	35	27	18
bedroom window open	“always”/“in summer”	11	17	11
	“never”	27	17	17
wish to leave bedroom window open	“yes”	24	24	17
	“no”	14	10	11
closing window because of noise	“never”	15	7	10
	at least sometimes	23	27	18
falling asleep difficult because of noise	“never”	29	29	23
	at least sometimes	9	5	5
awakenings by noise	“never”	23	25	17
	at least sometimes	15	9	11

### 3.2. Exposure Characteristics

**Figure 4:**
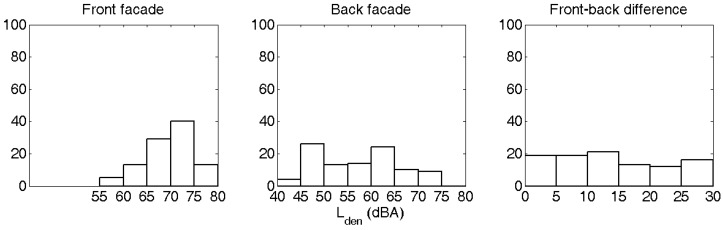
Exposure distribution at the front façade, back façade and absolute value of level difference at the survey locations.

The distribution of the front façade levels (near the street side) and back façade levels are shown in Figure 4 and Table 1. In 12% of the cases, the back façade noise level exceeds the front façade level. The distribution considering the most-exposed and least exposed façade is very similar (not shown). For 69% of the survey points, the most exposed façade falls between 65 and 75 dBA, which was the aim during site selection. Survey points outside this interval arise from the specific orientation and geometry of individual dwellings where someone was willing to participate. 38% of the respondents have a front-back level difference (absolute value) less than 10 dBA. 28% of the respondents have a level difference larger than or equal to 20 dBA.

The linear (Pearson’s) correlation coefficient between front level L_den_ and level difference equals 0.48 (95% CI = 0.31–0.62, *p* = 4E-7). This positive correlation is not surprising. Since minimum levels in the urban structure most often do not drop below a certain value, large level differences could only occur when the front façade level is high. The correlation coefficient found here is rather small, since the most exposed façade is fixed in a rather limited level interval during survey point selection, while locations are specifically searched for with a large and small level difference between front and back.

### 3.3. Analysis of Quality of the Living Environment, Annoyance and Quiet Side

#### 3.3.1. Quality of the Living Environment

**Figure 5:**
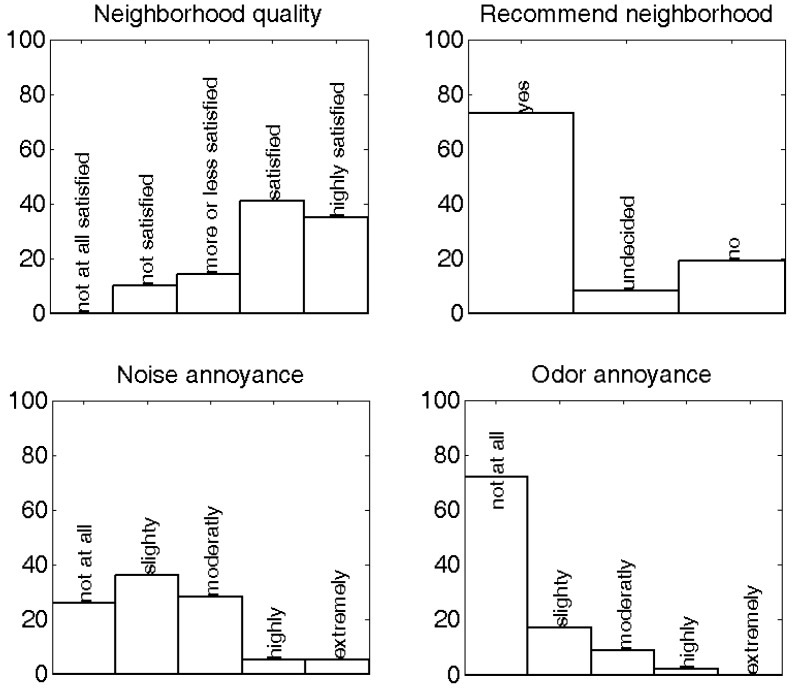
Distribution of answers of respondents related to neighborhood quality and different types of environmental nuisances.

An overview of the answers to questions on the quality of the living environment, and annoyance by different environmental stressors, is shown in Figure 5 and Table 1. Noise annoyance is strongly associated with self-reported satisfaction with the living quality of the neighborhood. People at least moderately annoyed (= “moderately”, “highly” and “extremely”) by noise are less satisfied (= “not” or “not at all” satisfied) with the quality of the neighborhood (OR = 4.4, 95% CI = 1.1–18.4). Independence of these variables can be strongly rejected (χ^2^ = 4.83, *p* = 0.03). There is a tendency that dwellers annoyed by noise would discourage relatives or friends to come live in their neighborhood, but this finding is not statistically significant (χ^2^ = 1.62, *p* = 0.20).

Odor annoyance has a much stronger impact on the living quality of the neighborhood (OR = 25.5; 95% CI = 5.4–120.6; χ^2^ = 27.25, *p* = 1.7E-7). Similarly, people annoyed by odor will not advise relatives or friends to come live in their neighborhood (χ^2^ = 13.11, *p* = 2.9E-4; OR = 9.8, 95% CI = 2.4–40.7). However, in the current dataset only 11% of the respondents are at least moderately annoyed by odor, and 57% of these cases comes from a single site characterized by industrial activities. Odor annoyance and noise annoyance in the current dataset were shown to be linked; their independence can be rejected with more than 90% certainty (χ^2^ = 3.45, *p* = 0.06). Further analysis on the latter will not be performed given the rather limited amount of cases with odor annoyance and the limited spread over the different sites in the current dataset. Furthermore, the interaction between these stressors can be complex, and there is evidence that noise and odor annoyance may strengthen each other when co-occurring [24].

#### 3.3.2. Noise Annoyance (in General)

Almost all reported noise annoyance could be attributed to road traffic noise, which is not surprising as all locations were selected based on the presence of rather high traffic noise levels at the most exposed façade. Noise annoyance and road traffic noise annoyance are strongly linked (χ^2^ = 43.36; *p* = 4.5E-11). The general question on noise annoyance revealed that 38% of the respondents were at least moderately annoyed; 10% was at least highly annoyed, and 5% of the respondents were extremely annoyed. The specific question on noise annoyance by road traffic noise showed that 45% of the respondents were at least moderately annoyed.

Noise annoyance caused by neighbors (at least “moderately annoyed”) was mentioned by 6% of the respondents. Neighbor noise annoyance is a variable constructed on the basis of the several sub-categories (see survey description) by using the maximum level of annoyance over all categories [25]. Other types of noise annoyance with a non-negligible frequency could be linked to road traffic noise: 7% were at least moderately annoyed by “street-racing”, 3% by “delivery of goods by vans”, and 3% by “loud music played in cars”. Annoyance by noise of ventilation units was not mentioned by any of the respondents. Given the fact that ventilation units are typically placed at the quiet side of a building, such noises could otherwise strongly deteriorate the quiet side soundscape.

The general noise annoyance question in this study was shown to be strongly independent of education (χ^2^ = 0.01, *p* = 0.92), independent of gender (χ^2^ = 0.13, *p* = 0.72), independent of years of living at the location (χ^2^ = 2.88, *p* = 0.58), and independent of age class (χ^2^ = 4.23, *p* = 0.38). In this analysis, education has been recoded to a dichotomous variable “continued education after secondary school”. Five age classes have been used (“16–30 years”, “31–40 years”, “41–50 years”, “51–60 years”, “more than 60 years”) to account for the expected non-monotonous dependence of annoyance on age [26]. The following classes related to years of living at the current locations were used: “1–2 years”, “2–5 years”, “5–15 years”, “15–30 years”, and “more than 30 years”. Analysis of large datasets shows no effect of gender [13], reduced noise annoyance at young and elderly people [26], and slightly higher annoyance with higher education level [13]. Given the limited dataset, these findings have not been found and were not intended to be studied here.

The averaged noise sensitivity rating (treated as a continuous variable) was shown to be positively correlated with noise annoyance (at least moderately annoyed) at an OR equal to 1.5 with the 95% CI = 1.0–2.3. However, independence testing (noise sensitivity recoded to a dichotomous variable, below or above a value of 3.5, which is the average between the endpoints of the proposed scale) did not give statistical significance with a sufficient degree of certitude (χ^2^ = 1.16, *p* = 0.28).

#### 3.3.3. Noise Annoyance in Relation to the Quiet Side Effect

The absence of a quiet façade at the dwelling (here defined as a level difference between front and back façade, Q, smaller than 10 dBA as predicted by the EU city noise map, as used in Reference [6]) leads to a statistically significantly higher chance of being at least moderately annoyed by road traffic noise at an OR equal to 2.7 (with 95% CI = 1.2–6.3). The absence of sufficient level difference between the most and least exposed façade, and noise annoyance are strongly dependent (χ^2^ = 5.57, *p* = 0.02). Only considering highly and extremely noise annoyed persons leads to a very similar OR, but not statistically different anymore from 1 giving the presence of only 10% of the samples falling in this class.

Noise sensitivity shows to be a modifier in relation to studying the effect of the absence of a quiet façade. Including noise sensitivity (continuous scale) in the logistic regression model significantly reduces the model deviance at the 5%-significance level, and both (independent) variable coefficients are significantly different from 0 at the 95% certainty level. The OR for at least moderate annoyance by street traffic noise in the absence of a quiet side increases from 2.7 (crude OR) to 3.3 (adjusted OR for noise sensitivity, with 95% CI = 1.3–8.0). Details on (multiple) logistic regression models, and related statistical parameters, are summarized in Table 2.

Inhabitants of dwellings with a pronounced quiet side (Q ≥ 20 dBA) double their chances to be “not at all” or only “slightly” annoyed by road traffic noise (OR = 2.3 with 95% CI = 0.9–6.0). This chance is not fully statistically significant. Note that in this level difference class (Q ≥ 20 dBA), there are less respondents than in the no-quiet side class (Q < 10 dBA). However, the hypothesis of independence between these two variables could be rejected at the 90% confidence level (χ^2^ = 2.79, *p* = 0.09). Only considering highly and extremely annoyed persons no longer leads to statistical significant conclusions. Similarly as with the no-quiet side analysis, accounting for noise sensitivity increases the OR, but only slightly and at the 10%-significance level. The adjusted OR now becomes 2.4 (with 95% CI = 0.9–6.4).

**Table 2:** Overview of logistic regression model statistics for at least moderately annoyed persons (dichotomous variable), awakening at least sometimes because of noise (dichotomous) and difficulties falling asleep at least sometimes because of noise (dichotomous). Only statistically significant model extensions (*p* < 0.10) have been considered. The logistic regression coefficients (beta), the standard errors (SE) on these variables, and the probabilities that model coefficients are equal to zero (*p*) are given together with their t-distribution values (*t*-value), odds-ratios (OR) and their 95% confidence intervals (95% CI on OR).

	beta	SE	*t*-value	*p*	OR	95% CI on OR
**Model output** **: at least moderately annoyed (1 = yes, 0 = no)**						
***MODEL1***						
cst	−0.201	0.225	−0.893	0.37		
bedroom at a quiet side (1 = yes, 0 = no)	−1.997	0.779	−2.565	0.01	1/7.39	[1/33.87, 1/1.57]
***MODEL2***						
cst	6.357	3.359	1.892	0.06		
bedroom at a quiet side (1 = yes, 0 = no)	−2.359	0.851	−2.773	0.01	1/10.58	[1/56.03, 1/2.00]
noise sensitivity (continuous)	−0.120	0.047	−2.532	0.01		
front level L_den_ (continuous)	0.449	0.257	1.748	0.08		
**Model output** **: at least moderately annoyed (1 = yes, 0 = no)**						
***MODEL1***						
cst	−0.894	0.280	−3.195	0.00		
Q < 10 (1 = yes, 0 = no)	0.999	0.429	2.330	0.02	2.72	[1.17, 6.29]
***MODEL2***						
cst	−2.973	1.020	−2.914	0.00		
Q < 10 (1 = yes, 0 = no)	1.190	0.455	2.619	0.01	3.29	[1.35, 8.01]
noise sensitivity (continuous)	0.511	0.235	2.176	0.03		
**Model output** **: at least moderately annoyed (1 = yes, 0 = no)**						
***MODEL1***						
cst	−0.280	0.238	−1.175	0.24		
Q ≥ 20 (1 = yes, 0 = no)	−0.819	0.497	−1.648	0.10	1/2.27	[1/6.01, 1/0.86]
***MODEL2***						
cst	−1.904	0.914	−2.083	0.04		
Q ≥ 20 (1 = yes, 0 = no)	−0.856	0.507	−1.688	0.09	1/2.35	[1/6.36, 1/0.87]
noise sensitivity (continuous)	0.417	0.224	1.862	0.06		
**Model output** **: awakenings at least sometimes by noise (1 = yes, 0 = no)**						
***MODEL***						
cst	−2.328	0.933	−2.496	0.01		
noise sensitivity (continuous)	0.435	0.227	1.916	0.06	1.54	[0.99, 2.41]
**Model output** **: difficulties falling asleep at least sometimes by noise (1 = yes, 0 = no)**						
***MODEL***						
cst	−1.237	0.268	−4.619	0.00		
bedroom at a quiet side (1 = yes, 0 = no)	−1.708	1.060	−1.611	0.11	1/5.52	[1/44.07, 1/0.69]

When Q is smaller than 10 dBA, this leads to a chance of 53% to be at least moderately annoyed (using model 1, see Table 2). For Q ≥ 20, this chance reduces to 25% (using model 1, see Table 2). Note that the level difference definitions are rather rough, taking into account the expected inaccuracies in the calculation methodologies applied to produce city-wide noise maps (see earlier discussion). Another reason for this coarse classification is to end up with a sufficient number of occurrences for each variable combination in this dichotomous approach, giving the limited dataset. Further refinement is therefore not made. Not surprisingly, the intermediate level difference class (between 10 dBA and 20 dBA) did not give statistically significant findings.

Front façade noise level (or the most exposed façade level) and level difference are linearly correlated as shown during the exposure analysis. However, including front or back façade L_den_ in the logistic regression model does not give a significant improvement of the model or model coefficients statistically different from 0, even when allowing for a large degree of uncertainty. Note that the range of front-level exposure is kept deliberately limited in this study, allowing to focus on the effect of a quiet side. The reader should keep in mind that front façade level is most likely to be the most important independent variable when considering a broad range of L_den_ values, as classically used in exposure-effect relationships (see e.g., Reference [27]).

### 3.4. Sleep Disturbance and Quiet Side

**Figure 6:**
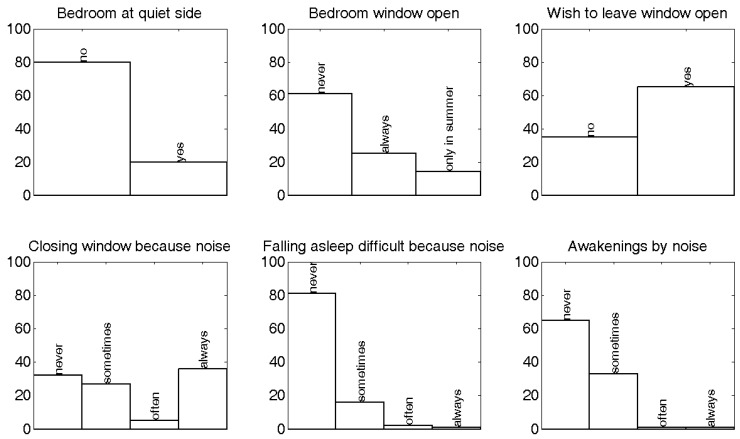
Distribution of answers of respondents related to sleep disturbance and bedroom window use. The assessment whether the bedroom was located at the quiet side was made by the interviewer.

An overview of the answers to questions on sleep disturbance, and window use and habits, is shown in Figure 6 and Table 1.

It was objectively observed by the interviewer that only 20% of the respondents have their bedroom window at a quiet façade. Persons having their bedroom at a quiet façade have 7.4 times (crude OR, 95% CI = 1.6–33.9) more chance of not being (at least moderately) annoyed by noise compared to the 80% of the respondents having their bedroom at an exposed side. This dependence is highly statistically significant (χ^2^ = 8.3, *p* = 0.004). The predicted chance of being at least moderately annoyed by noise (using model 1, see Table 2) reduces from 45% (bedroom not at a quiet side) to 10% when the bedroom is located at a quiet side. This indirect effect of bedroom location on noise annoyance rating seems much stronger than the direct effect of level difference Q as quantified in previous section.

Including noise sensitivity and front-façade L_den_ level (see Table 2) in the logistic regression model significantly reduces model deviation (at the 5% significance level). The adjusted OR for bedroom location increases to 10.6 (with 95% CI = 2.0–56.0). It can therefore be concluded that the location of the sleeping room at a quiet side has an important (indirect) positive effect on the (general) noise annoyance experienced at the dwelling. This indirect quiet side mechanism seems especially relevant for noise sensitive persons. Among the highly and extremely annoyed people, no one in the current sample had the bedroom window facing the quiet side.

61% of the respondents close their bedroom window during sleeping. The answers “always open”, and “only during summer period” have been grouped to the same category in the following analysis. The fact that 80% of the respondents have their bedroom near the loudest side already suggests a logical link. The people closing their window are not happy with this; near one half (46%) of them would actually prefer to sleep with open windows. The question whether they close their bedroom window because of road traffic noise was answered affirmative (closing “sometimes”, “often”, or “always” the window) by 74% of the people. Preference with regards to opening or closing window, and closing because of road traffic noise, tend to be dependent (χ^2^ = 1.88, *p* = 0.17). People closing their windows because of noise have an OR of 2.3 (95% CI = 0.7–7.7) that they do this against their actual preference as for closing windows. This is however not statistically significant at the 95% certainty level. Only 8% of the people closing their window, and doing this against their will, seem to have another reason than road traffic noise. Noise sensitive persons close their bedroom window more often because of road traffic noise. Noise sensitivity classified as a dichotomous variable (below or above 3.5) and closing bedroom window because of noise (closing “sometimes”, “often” or “always” versus “never”) yields a strong dependency (χ^2^ = 6.38, *p* = 0.01; OR = 3.1 with 95% CI = 1.3–7.7).

Persons whose sleeping room is located at the quiet side of a dwelling, have a chance that is 19 times (95% CI = 2.4–148.8) as low to close their window because of street noise than people with a bedroom at a loud side. This dependence is highly statistically significant (χ^2^ = 13.39, *p* = 2.5E-4). People sleeping with open window do this fully following their preference (OR = 21.8 with 95% CI = 4.8–98.6). This is in contrast with people closing bedroom windows, which is often against their wishes. The latter can be seen as a way of active coping with excessive noise levels. People create their own quiet side when the rooms are not well orientated. This analysis is however somewhat biased, since people having their sleeping room at a quiet side have a somewhat stronger preference to sleep with open window, but independency between these variables cannot be statistically significantly rejected at the 5%-significance level (χ^2^ = 2.47, *p* = 0.12). A possible reason is that while choosing bedroom location after moving or during designing a newly built house, this preference could have been taken into account. Furthermore, bedroom location at a quiet side and noise sensitivity are moderately dependent (χ^2^ = 2.68, *p* = 0.10): noise sensitive people seem to have taken noise into account when making such decisions.

38% of the people with a bedroom at a loud side (both closing and not-closing windows) wake up at least sometimes because of street noise. This percentage reduces to 25% for people with a bedroom at a quiet side. However, awakening (“sometimes”, “often” or “always”) at night by road traffic noise does not seem to be strongly dependent on the location of the bedroom at a quiet side (χ^2^ = 1.10, *p* = 0.29; OR = 1.8 with 95% CI = 0.6–5.5). Noise sensitivity (continuous scale) is here the variable with most predictive power (see Table 2) among the ones considered, at an OR of 1.5 (with 95% CI = 1.0–2.4). Classifying noise sensitivity to a dichotomous variable (below or above a value of 3.5) shows some dependency (χ^2^ = 2.65, *p* = 0.11) with self-reported awakening.

Difficulties falling asleep because of noise and the presence of the bedroom at the quiet side are somewhat more significantly dependent (independence can be rejected with 93% certainty following the chi-square test, χ^2^ = 3.18, *p* = 0.07). A sleeping room at the quiet side gives an OR equal to 5.5 (95% CI = 0.7–44.1) in preventing to have (at least sometimes) difficulties falling asleep because of noise. The predicted chance of having difficulties falling asleep at least sometimes by noise reduces from 23% (bedroom not at a quiet side) to 5% when the bedroom is located at a quiet side. Noise sensitivity is not an important parameter for the latter, and hardly improves model accuracy when adding this parameter as a second independent variable in the logistic regression.

Awakening at night by noise and having problems falling asleep because of noise are clearly linked (χ^2^ = 19.9, *p* < 1E-5; OR = 11.4 with 95% CI = 3.4–38.5). Awakening by street noise strongly influences the (general) noise annoyance experienced. Waking up at least sometimes by noise, and being at least moderately annoyed, both classified as a dichotomous variable, are strongly dependent (χ^2^ = 8.38, *p* = 0.004; OR = 3.5 with 95% CI = 1.5–8.2). The link between difficulties falling asleep because of street noise and general noise annoyance is not at all present.

## 4. Conclusions

This study aimed at providing additional evidence related to the quiet side effect by means of a face-to-face questionnaire and to unravel potential mechanisms to explain the quiet side effect. Despite the rather limited number of respondents, decreasing statistical power, statistically significant findings could be reported. An important condition in such a case is control of the noise level at the most exposed façade, which is a decisive parameter to predict noise annoyance [26]. The current study is focused on a group of dwellings with high front façade L_den_ traffic noise levels within a narrow interval (near dense, non-highway road traffic). This allowed us to limit any variability in effects that might be caused by exposure at the front façade. In addition, a broad range of back façade levels were deliberately looked for to increase contrast (no quiet side at all versus a highly shielded façade). As a consequence, different types of neighborhoods were considered. Structural differences in dwelling characteristics in between neighborhoods could not be excluded like e.g., noise insulation quality of facades or dwelling size.

It was assured that the back façade was accessible at least by windows facing it, and that no other sound sources were present, except for occasional neighbor noise. Not surprisingly, annoyance by road traffic noise was clearly the most important and only source of noise annoyance.

The absence of a quiet side (here considered as a level difference less than 10 dBA between most and least-exposed façade) leads to a significantly higher chance of being more annoyed by noise. A large level difference (at least 20 dBA) leads to a lower chance of begin annoyed, however, somewhat less statistically significant than this first finding, most likely because of less survey points obeying this condition. The chance of being at least moderately annoyed reduces from 53% to 25% when going from Q < 10 dBA to Q ≥ 20 dBA.

An indirect effect on self-reported noise annoyance occurs via the bedroom location, and was shown to be even more important than the direct effect of level difference. Noise-induced sleep disturbance, especially awakenings at night by noise, is strongly associated with self-reported noise annoyance. This indirect quiet side effect seems to be especially important for noise-sensitive persons. Also for the direct effect, noise sensitivity is a statistically relevant effect modifier, however, influencing the odds-ratio less strongly. A bedroom located at a quiet side also has a direct influence on noise-induced sleep disturbance: it leads to less (self-reported) awakenings and allows falling asleep more easily.

Noise sensitive persons cope with excessive noise exposure: they close the bedroom window more often because of road traffic noise, and their bedroom is more often located at the quiet side of the dwelling compared to persons that are less sensitive to noise. When the bedroom is located at the loud side, closing windows because of road traffic noise often conflicts with the actual wish of the dwellers.

The current study relies on a city-wide official noise map from which the sound level predictions at a quiet facade are known to be of rather limited accuracy. Therefore, only a rough classification regarding level difference between the loudest and quietest side was used during analysis
